# LINC00641/miR-582-5p mediate oxaliplatin resistance by activating autophagy in gastric adenocarcinoma

**DOI:** 10.1038/s41598-020-70913-2

**Published:** 2020-09-11

**Authors:** Yunfeng Hu, Yani Su, Xia Lei, Hong Zhao, Lelin Wang, Tian Xu, Jing Guo, Weiwei Yang, Xiaozhi Zhang

**Affiliations:** 1 Department of Radiation Oncology, The First Affiliated Hospital of Xi’an Jiaotong University, Xi’an, Shaanxi China; 2 Department of Radiation Oncology, The First Affiliated Hospital of Yan’an University, Yan’an, Shaanxi China; 3 Department of Gynecology, The First Affiliated Hospital of Yan’an University, Yan’an, Shaanxi China; 4 Department of Thoracic Surgery, The First Affiliated Hospital of Xi’an Jiaotong University, Xi’an, Shaanxi China

**Keywords:** Molecular biology, Cancer, Gastrointestinal cancer

## Abstract

The poor prognosis of gastric adenocarcinoma is partly due to chemotherapy failure, especially the oxaliplatin-based chemotherapy. However, the specific mechanism of oxaliplatin resistance is unclear. We aim to find the roles that LINC00641 and miR-582-5p play in regulating oxaliplatin resistance. Quantitative reverse transcriptase-PCR was used to evaluate the expression of LINC00641 and microRNA-582-5p (miR-582-5p) in gastric cancer both in vivo and in vitro. Transwell and CCK-8 assays were performed; and LC3 I/II and p62 were detected by western blot to evaluate the activation of autophagy. LINC00641 expression was associated with prognosis and oxaliplatin resistance in patients with gastric adenocarcinoma. The expression of LINC00641 was higher in gastric cancer tissues; whereas miR-582-5p was down-regulated in gastric cancer tissues. Moreover, LINC00641 was highly expressed in oxaliplatin-resistant cell lines and miR-582-5p was down-regulated. In addition, LINC00641 negatively regulated the expression of miR-582-5p. With regard to biological functions, down-regulation of LINC00641 suppressed cell migration and proliferation. Further experiments indicated that down-regulation of LINC00641 inhibited the autophagy process, making gastric cancer cells more sensitive to oxaliplatin. LINC00641 and miR-582-5p are biomarkers for predicting overall survival, as they were involved in regulating oxaliplatin resistance by altering autophagy in gastric adenocarcinoma.

## Introduction

Gastric carcinoma is the fifth most common cancer worldwide and is the third leading cause of cancer death^1^. Although the incidence and mortality rates of gastric adenocarcinoma have continued to decline in recent years, the mortality rate is still high in China^2^. Due to a lack of typical and specific symptoms, many patients are diagnosed at advanced stages with distant metastases, and are often unable to undergo surgical resection. The only treatment option for these patients is chemotherapy. In gastric carcinoma, oxaliplatin (L-OHP) is one of the most important anti-cancer agents^3^. However, L-OHP resistance frequently occurs during treatment and significantly limits the efficacy of L-OHP^4^. In this context, it is necessary to investigate the molecular mechanisms of L-OHP resistance in gastric adenocarcinoma, and identify effective therapeutic targets.

Autophagy is a biological process, where cells capture and degrade damaged intracellular organelles and proteins by fusing them with lysosomes. Autophagy enables cells to maintain metabolism and survival by recycling intracellular proteins to cope with stress^5^. Therefore, it has been suggested that autophagy may be involved in chemo-resistance. Numerous studies have indicated that autophagy can be induced by DNA damage, which can be caused by ionizing or anticancer agents^6,7^. Previous studies found that ATM, a DNA damage sensor, could activate TSC2 via the LKB1/AMPK pathway and then induce autophagy^8^. Based on these results, the inhibition of autophagy may be a promising strategy to increase tumor chemotherapy sensitivity.

Many biological functions of long non-coding RNA (lncRNA) in cancer have attracted significant attention in recent years. LncRNA represents a group of RNA transcripts that are longer than 200 nucleotides but cannot be converted into proteins^9^. A number of studies have shown that lncRNAs are involved in the initiation of autophagy. It was previously demonstrated that lncRNA H19 could be down-regulated in a high glucose environment and then promote the expression of DIRAS3. High expression of DIRAS3 leads to the initiation of autophagy through inhibition of the PI3K/AKT/mTOR pathway^10^. Among the various lncRNAs, a novel lncRNA, named LINC00641, has attracted our attention. A previous study indicated that LINC00641 could act as the competing endogenous RNA (ceRNA) by binding microRNA-153-3p (miR-153-3p) to relieve the transcriptional suppression of ATG5, and then promote the initiation of autophagy during intervertebral disc degeneration^11^. However, the biological function of LINC00641 in carcinoma is still unclear. Only one study has reported that LINC00641 expression was down-regulated in bladder cancer, and it could suppress the progression of bladder cancer via the miR-197-3p/KLF10/PTEN/PI3K/AKT pathway^12^. However, whether LINC00641 participates in the regulation of autophagy in gastric carcinoma is still unknown. In this study, we found that LINC00641 was highly expressed in gastric carcinoma, and up-regulated expression of LINC00641 was associated with a poor prognosis in patients with gastric carcinoma. We then investigated the interaction between LINC00641 and autophagy in gastric carcinoma and found that LINC00641 could mediate L-OHP resistance in gastric carcinoma by regulating autophagy initiation.

## Materials and methods

### Patients

One hundred and seventy-three gastric cancer tissues and corresponding normal tissues were obtained from the First Affiliated Hospital of Yan’an University. All patients received capecitabine plus L-OHP (XELOX) chemotherapy after surgery. These patients did not receive any other treatment for other diseases during chemotherapy. We have performed regular imageological examination for every patient every 3 month for 1 year. The recurrent patients were classified as XELOX resistant patients. All patients provided a written informed consent and the Ethics Committee of the First Affiliated Hospital of Yan’an University approved the study. This study was performed in accordance with the Helsinki declaration.

### Cell culture, transfection and establishment of an L-OHP resistant cell line

MKN45, SGC7901 and MKN28 cells were purchased from American Type Culture Collection (ATCC, Manassas, VA, USA) and were cultured in RPMI 1640 medium and 10% fetal bovine serum (FBS) (Gibco, USA). The incubation environment consisted of 37 ℃ and 5% CO_2_.The LINC00641 down-regulating lentivirus, miR-582-5p down-regulating lentivirus, miR-582-5p up-regulating lentivirus and relevant negative control lentiviruses were purchased from GeneChem (China). The transfection process was completed according to the manufacturer's guidelines. hU6-MCSUbiquitin-EGFP-IRES-puromycin was used as the vector for the miR-582-5p overexpression lentivirus; hU6-MCSCMV-EGFP was used as the vector for the LINC00641 and miR-582-5p knockdown lentiviruses.

The oxaliplatin-resistant SGC-7901 cell line was named LOHPR SGC7901. SGC-7901 was treated with 1 mL 2 µg/mL oxaliplatin for 1 day; and then change the medium into L-OHP free medium for 1 day. This process would be repeated until the SGC7901 cell growth was not influenced by L-OHP. After that, the L-OHP concentration would be 3 µg/mL, 4 µg/mL, 6 µg/mL, 8 µg/mL, and 10 µg/mL. SGC-7901 cells that can survive with 10 µg/mL L-OHP were considered as L-OHP resistant cells.

### Quantitative reverse transcriptase PCR (qRT-PCR)

Total RNA was extracted using Trizol reagent (Takara, Japan). PrimeScriptTM RT Master Mix purchased from Takara and the Taqman miRNA reverse transcription Kit (Carlsbad, USA) were used for reverse-transcription. SYBR Select Master Mix was used for qPCR. The relative expression of LINC00641 and miR-582-5p were normalized to GAPDH and U6. The primers are as follows:

LINC00641:

Forward 5′-CAGCCTATGACAGACAGCCC-3′

Reverse 5′-CCAGTTGGTGCTGCCATTTG-3`

miR582-5p:

Forward 5′-ATCCCTAGCTTCAACGTG-3′

Reverse 5′-CGTTACAATTGCTAGC-3′

U6:

Forward 5′-CTCGCTTCGGCAGCACA-3′

Reverse5′-AACGCTTCACGAATTTGCGT-3′

GAPDH:

Forward 5′-ACAACTTTGGTATCGTGGAAGG-3′

Reverse 5′-GCCATCACGCCACAGTTTC-3′

### Western blot

Total protein was extracted using RIPA buffer (Sigma-Aldrich, MA, USA); and we used BCA (Sigma-Aldrich, MA, USA) to validate the protein extracted. The extracted protein samples were electrophoresed by 10% SDS-PAGE. Proteins were then transferred onto a polyvinylidene fluoride membrane and were blocked with 5% nonfat milk for 2 h at room temperature. Primary antibodies were then incubated with the membrane overnight. After that, secondary antibodies (1:3,000, CST; rabbit 7074) were incubated for 1 h. Electrochemiluminescence was used to visualize the western blots. The primary antibodies of LC3 I/II (1:1,000, 23214) , p62 (1:1,000, 4108S) and GAPDH (1:1,000, 5174) were purchased from CST.

### Transwell assay

Cells were cultured in FBS-free medium for 24 h. 5 × 10^4^ cells were placed in the upper chamber in FBS-free medium. After 24 h, migrated cells were stained using crystal violet. Images were then obtained with a light microscope at 200× magnification.

### Cell proliferation test

Cell counting kit-8 (CCK-8) (Dojindo, Japan) was used to evaluate cell proliferation. Cells were seeded into 96-well plates at a density of 1,000 cells per well and 10 μL CCK8 reagent was added before evaluation. The absorbance was measured at 24, 48 and 72 h at a wavelength of 450 nm.

### Dual luciferase reporter gene assay

pmirGLO, pmirGLO-linc00641-WT and pmirGLO-linc00641-Mut (miR-582-5p) were purchased from Genchem, The sequences of the constructed plasmids and miRNAs were confirmed by DNA sequencing (Sangon Biotech, China). A luciferase assay was performed 24 h after transfection using the Dual‐Luciferase Assay System (Promega, China) according to the manufacturer's protocol. Luciferase activity was normalized to Renilla luciferase activity.

### RNA pull-down assay

The biotinylated probes miR-582-5p-Wt and miR-582-5p-Mut were synthesized by Genecreate (Wuhan, China), which was transfected into SGC7901. M-280 Streptavidin-coated MagneSphere particles were used to bind to the biotinylated RNA. Then PBS was used for elution the RNA, which was then harvested and purified. The harvested RNA was reverse-transcribed into cDNA and qRT-PCR was used to investigate the expression of OIP5-AS1.

### Analysis of autophagy

Cells were transfected with the SensGFP-StubRFP-LC3 virus, which was constructed by Genechem. After 24 h of transfection, cells were selected with puromycin. Cells (1 × 10^4^) were plated into 96-well plates and scanned using a Confocal Quantitative Image cytometer (YOKOGAWA, Tokyo, Japan).

### Statistical analysis

The Student’s *t *test and Chi-square tests were adopted to compare the differences between two groups. A *P* value < 0.05 was considered statistically significant. Nomograms were constructed based on selected variables from a Cox regression model to predict 1-year and 3-year survival rates. The C-index was calculated to test the discrimination of the nomogram with 1,000 bootstrap resamples. In addition, calibration curves were plotted for nomogram calibrations, where distance from the calibration curve to the 45° straight indicated the precision. All statistical analyses were performed with R 3.3.5 software. All figures were plotted or processed using Prism software.

## Results

### LINC00641 expression was correlated with L-OHP resistance in gastric cancer patients

**Figure 1:**
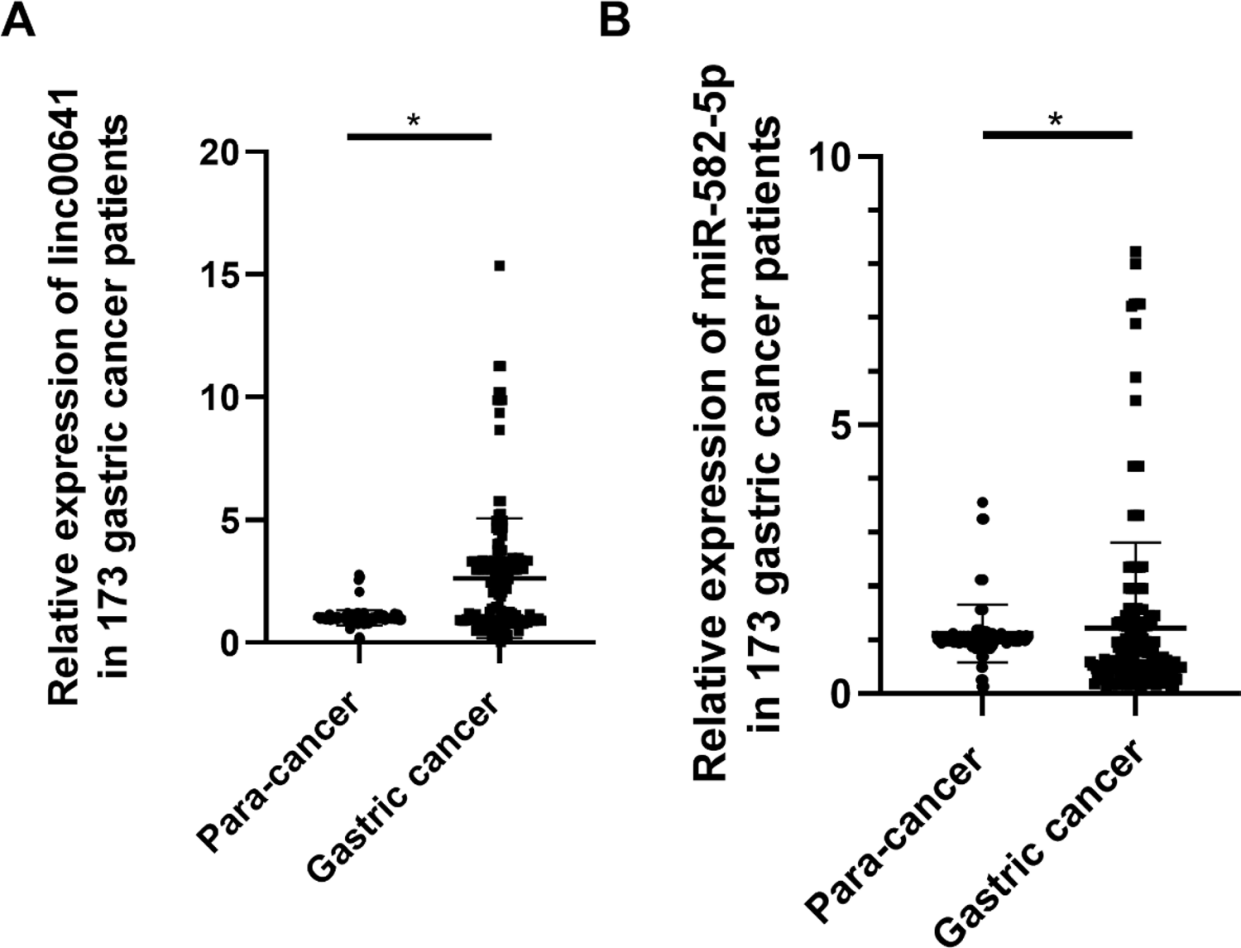
(**A**) The expression of LINC00641 in 173 gastric cancer patients; (**B**) the expression of miR-582-5p in 173 gastric cancer patients.

LINC00641 was highly expressed in 173 gastric cancer tissues compared with para-cancer tissues, and miR-582-5p expression was lower in cancer tissues (Fig. 1A,B). Therefore, the patients were grouped into the LINC00641 high expression group and the LINC00641 low expression group. These results showed that LINC00641 expression correlated with L-OHP resistance, miR-582-5p expression, M stage and patient sex (Table 1). Thus, we assumed that LINC00641 might be involved in the process of L-OHP resistance by negatively regulating miR-582-5p expression.

**Table 1:** The relation of LINC00641 expression between clinical variables.

Variables	N	Low expression (Linc00641, N = 62)	High expression (Linc00641, N = 111)	P value
**Age**				0.761
< 60	78	27	51	
≥ 60	95	35	60	
**Sex**				0.034
Female	80	22	58	
Male	93	40	53	
**T stage**				0.164
T1	86	36	50	
T2	42	16	26	
T3	35	8	27	
T4	10	2	8	
**N stage**				0.895
N0	47	15	35	
N1	78	30	48	
N2	30	11	19	
N3	18	6	12	
**M stage**				0.008
M0	121	51	70	
M1	52	11	41	
**L-OHP resistance**				0.017
No	108	46	62	
Yes	65	16	49	
**CEA**				0.196
Low expression	109	43	66	
High expression	64	19	45	
**CA724**				0.289
Low expression	136	46	90	
High expression	37	16	21	
**CA199**				0.299
Low expression	128	43	85	
High expression	45	19	26	
**miR-582-5p**				0.022
Low expression	114	34	80	
High expression	59	28	31	

P values were calculated by Chi-square test.

### Nomogram for LINC00641/miR-582-5p in gastric cancer patients

We performed log-rank tests and found that LINC00641, miR-582-5p, M stage, L-OHP resistance, carcinoembryonic antigen (CEA) and patient age were associated with prognosis (Table 2).

**Table 2:** Survival analysis for clinical manifestations in gastric cancer patients.

Variables	Survival months	95% CI of survival months		P value
		Lower 95% CI	Upper 95% CI	
**Age**				0.019
< 60	36.210 ± 2.693	30.931	41.489	
≥ 60	28.411 ± 2.496	23.518	33.304	
**Sex**				0.689
Female	28.333 ± 2.185	24.051	32.615	
Male	32.655 ± 2.594	27.570	37.740	
**T stage**				0.147
T1	35.109 ± 2.495	30.219	39.998	
T2	30.233 ± 4.107	22.183	38.284	
T3	25.366 ± 3.397	18.708	32.024	
T4	16.567 ± 3.261	10.175	22.958	
**N stage**				0.340
N0	25.072 ± 3.026	19.142	31.002	
N1	33.905 ± 2.642	28.727	39.084	
N2	32.669 ± 4.789	23.281	42.056	
N3	31.208 ± 5.986	19.475	42.942	
**M stage**				0.000
M0	38.672 ± 2.056	34.641	42.702	
M1	15.435 ± 2.850	9.849	21.022	
**L-OHP resistance**				
No				
Yes				
**CEA**				0.024
Low expression	34.886 ± 2.365	30.252	39.521	
High expression	26.789 ± 2.867	21.169	32.409	
**CA724**				
Low expression	31.546 ± 2.126	27.380	35.712	
High expression	32.878 ± 3.921	25.192	40.563	
**CA199**				0.379
Low expression	30.732 ± 2.140	26.537	34.928	
High expression	34.211 ± 3.624	27.108	41.314	
**miR-582-5p**				0.006
Low expression	27.500 ± 2.171	23.244	31.755	
High expression	39.124 ± 3.276	32.703	45.545	
**Linc00641**				0.037
Low expression	37.249 ± 3.064	31.244	43.255	
High expression	28.485 ± 2.311	23.957	33.014	

P values were calculated by log-rank test.

**Figure 2:**
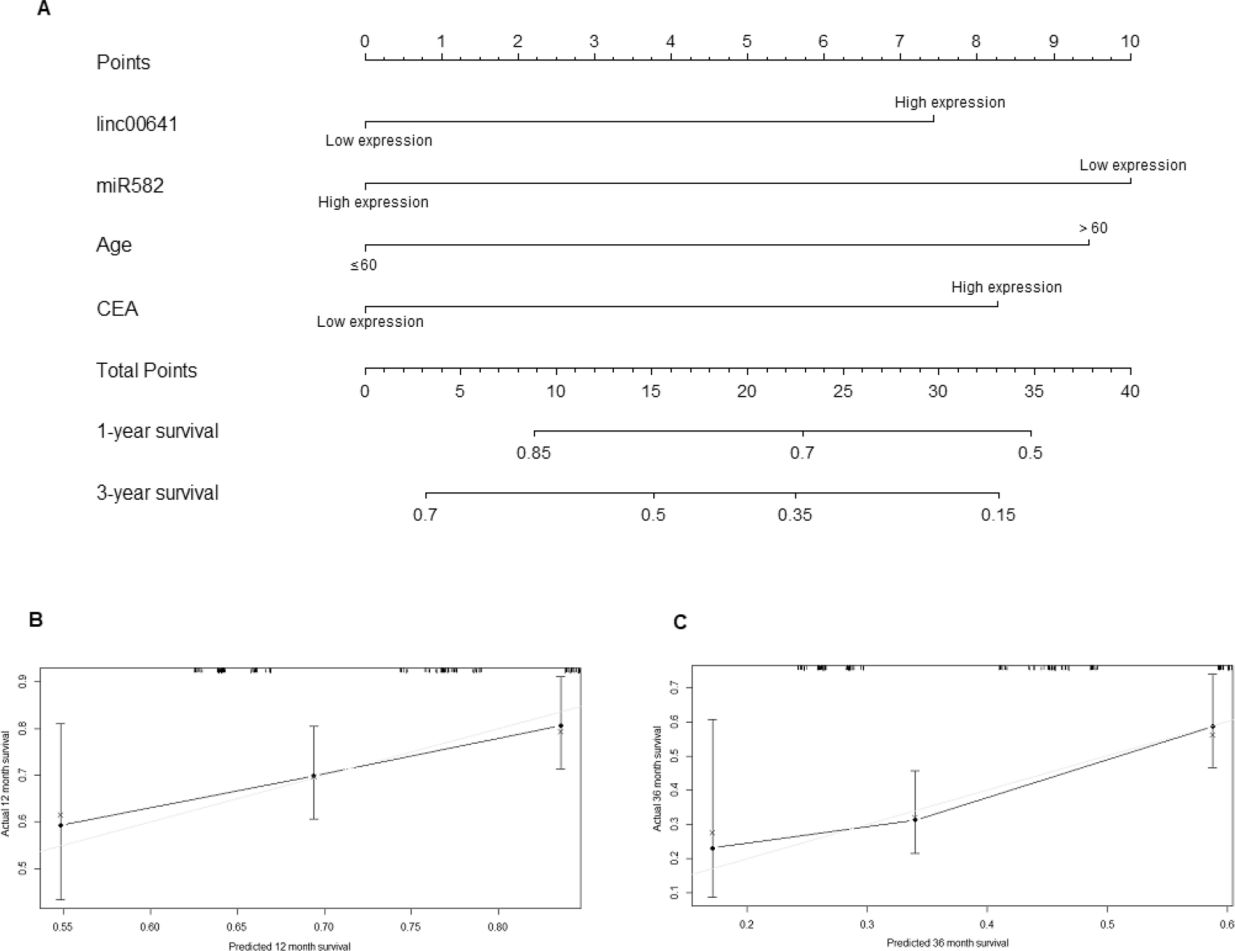
(**A**) Nomogram for LINC00641, miR-582-5p, age and CEA. (**B**) 1-Year internal calibration curve for the nomogram. (**C**) 3-Year internal calibration curve for the nomogram.

We then used the COX regression model for variables including LINC00641, miR-582-5p, CEA and age (Supplementary Table 1), and established a nomogram to visualize the COX regression model (Fig. 2A). The calibration curves indicated that this nomogram could predict the 1-year and 3-year survival rates in gastric cancer patients (Fig. 2B,C). In addition, the C-index was 0.876 ± 0.06, which indicated that the nomogram could predict the survival rate accurately and precisely. Thus, LINC00641 and miR-582-5p could be biomarkers for predicting overall survival rate in gastric cancer patients.

### LINC00641 promoted cell proliferation and migration by inhibiting miR-582-5p in vitro

**Figure 3:**
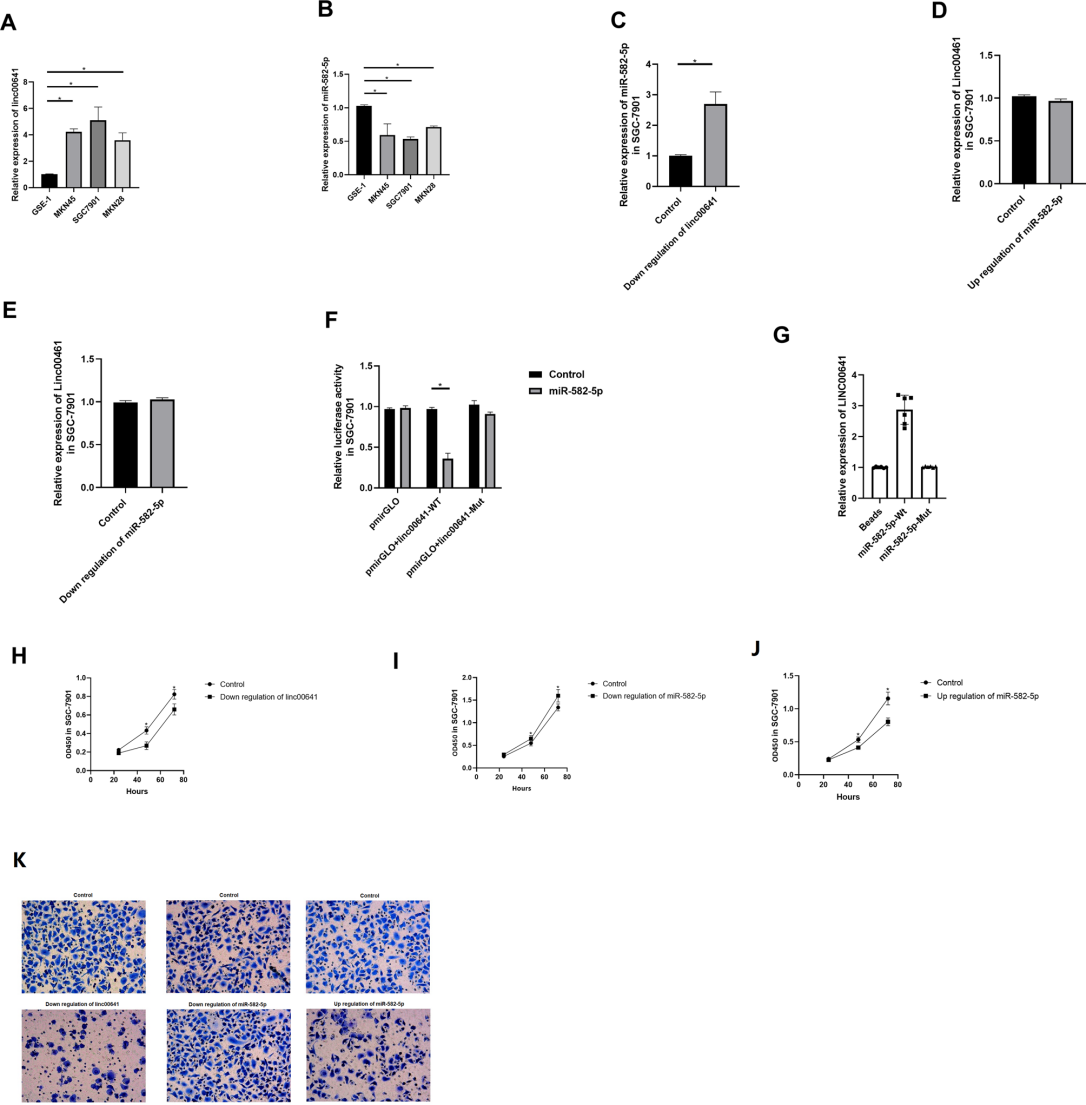
(**A**) The expression of LINC00641 in gastric cancer cell lines; (**B**) the expression of miR-582-5p in gastric cancer cell lines; (**C**) the expression of miR-582-5p in LINC00641 knock down SGC-7901 cell line; (**D**) the expression of LINC00641 in miR-582-5p overexpression SGC-7901 cell line; (**E**) the expression of LINC00641 in miR-582-5p knock down SGC-7901 cell line; (**F**) dual luciferase reporter gene assay to investigate the interaction of LINC00641 and miR-582-5p; (**G**) RNA pull down assay to investigate the interaction of LINC00641 and miR-582-5p; (**H**) CCK-8 assay to investigate the proliferation ability in LINC00641 knock down SGC-7901 cell line; (**I**) CCK-8 assay to investigate the proliferation ability in miR-582-5p knock down SGC-7901 cell line; (**J**) CCK-8 assay to investigate the proliferation ability in miR-582-5p overexpression SGC-7901 cell line; (**K**) transwell assay to investigate the migration ability. Images were photographed at ×200.

In gastric cancer cell lines, LINC00641 was highly expressed in MKN45, MKN28 and SGC7901 cancer cells compared with normal GES-1 gastric cells. The expression of miR-582-5p in these cancer cells was low (Fig. 3A,B). We then down-regulated the expression of LINC00641 in the SGC7901 cell line and found that the expression of miR-582-5p was inhibited (Fig. 3C). When the expression of miR-582-5p was up- and down-regulated, the expression of LINC00641 was unaffected (Fig. 3D,E). In this context, we assumed that miR-582-5p was the downstream target of LINC00641. Therefore, we performed a dual luciferase reporter gene assay and found that miR-582-5p interacted with the 3′UTR of LINC00641 (Fig. 3F). Moreover, the interaction was then confirmed by RNA-pull down assay: LINC00641 was significantly expressed higher in miR-582-5p-Wt group than that in miR-582-5p-Mut group (Fig. 3G).

With regard to biological functions, the CCK-8 assay was performed and the results showed that down-regulation of LINC00641 led to decreased cell proliferation in the SGC7901 cell line (Fig. 3G). It was also found that down-regulation of miR-582-5p led to increased cell proliferation, whereas up-regulation of miR-582-5p resulted in decreased cell proliferation (Fig. 3H,I). In the transwell assay, down-regulation of LINC00641 was associated with decreased migration rate (Fig. 3J). Down-regulation of miR-582-5p resulted in increased migration rate and up-regulation of miR-582-5p resulted in decreased migration rate (Fig. 3K).

### LINC00641 and miR-582-5p were aberrantly expressed in L-OHP resistant gastric cancer cells

**Figure 4:**
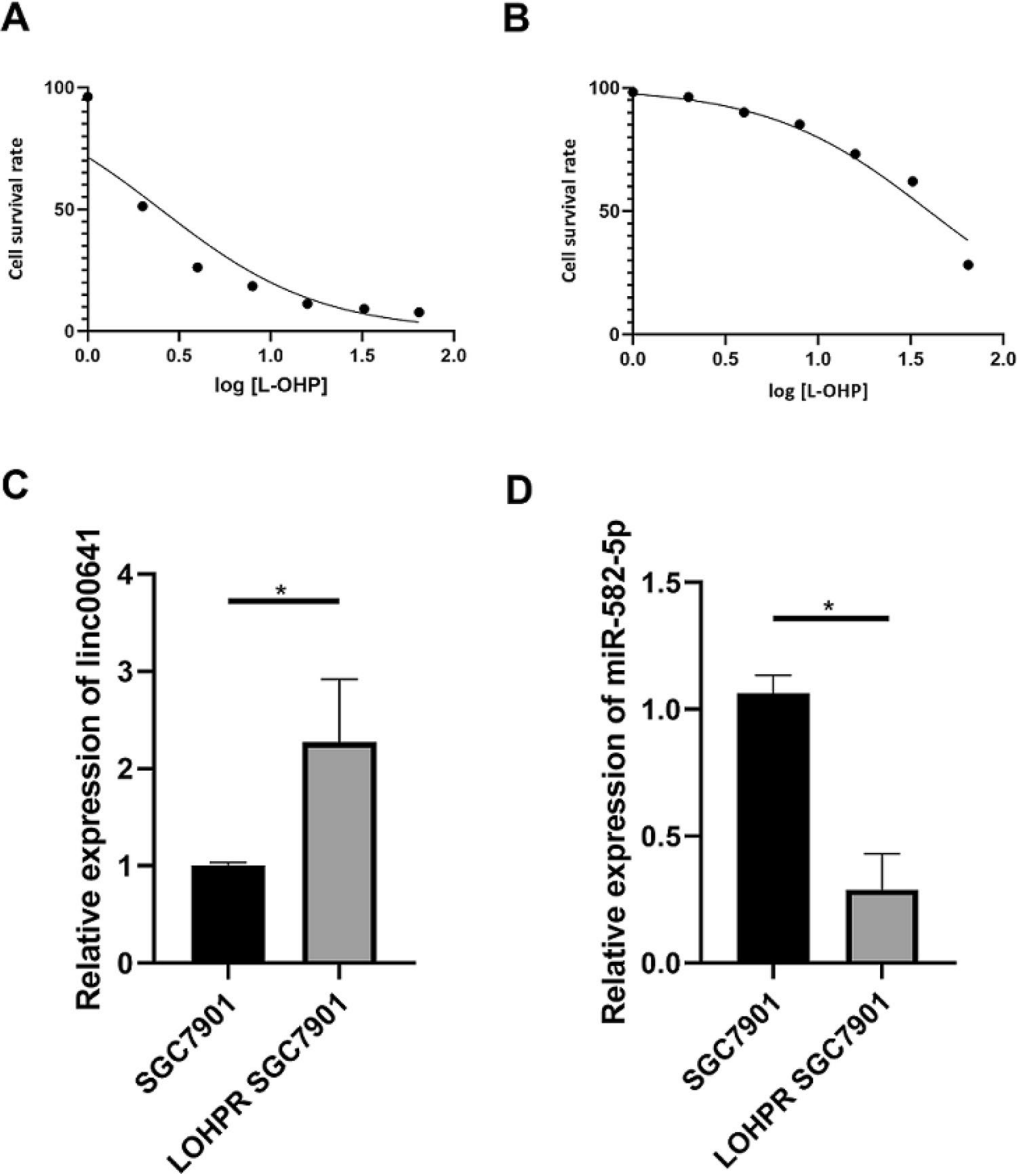
(**A**) Cell survival rate for SGC7901 cell treated with different L-OHP concentration; (**B**) cell survival rate for L-OHPR SGC7901 cell treated with different L-OHP concentration; (**C**) the expression of LINC00641 in LOHPR SGC7901 cell line compared with SGC7901 cell line; (**D**) the expression of miR-582-5p in LOHPR SGC7901 cell line compared with SGC7901 cell line.

We used a range of L-OHP concentrations to treat the SGC7901 and L-OHP-resistant SGC7901 cell lines; and the results showed that the half maximal inhibitory concentration (IC50) for SGC7901 cells was 2.51 and the IC50 for L-OHP-resistant SGC7901 cells was 39.79 (Fig. 4A,B). Therefore, the L-OHP resistance index was 15.42, indicating that L-OHP-resistant SGC7901 cells could tolerate L-OHP well. We also found that LINC00641 was highly expressed in the L-OHP-resistant SGC7901 cell line and the expression of miR-582-5p was lower compared with SGC7901 cells (Fig. 4C,D).

### LINC00641 regulates the autophagy process

**Figure 5:**
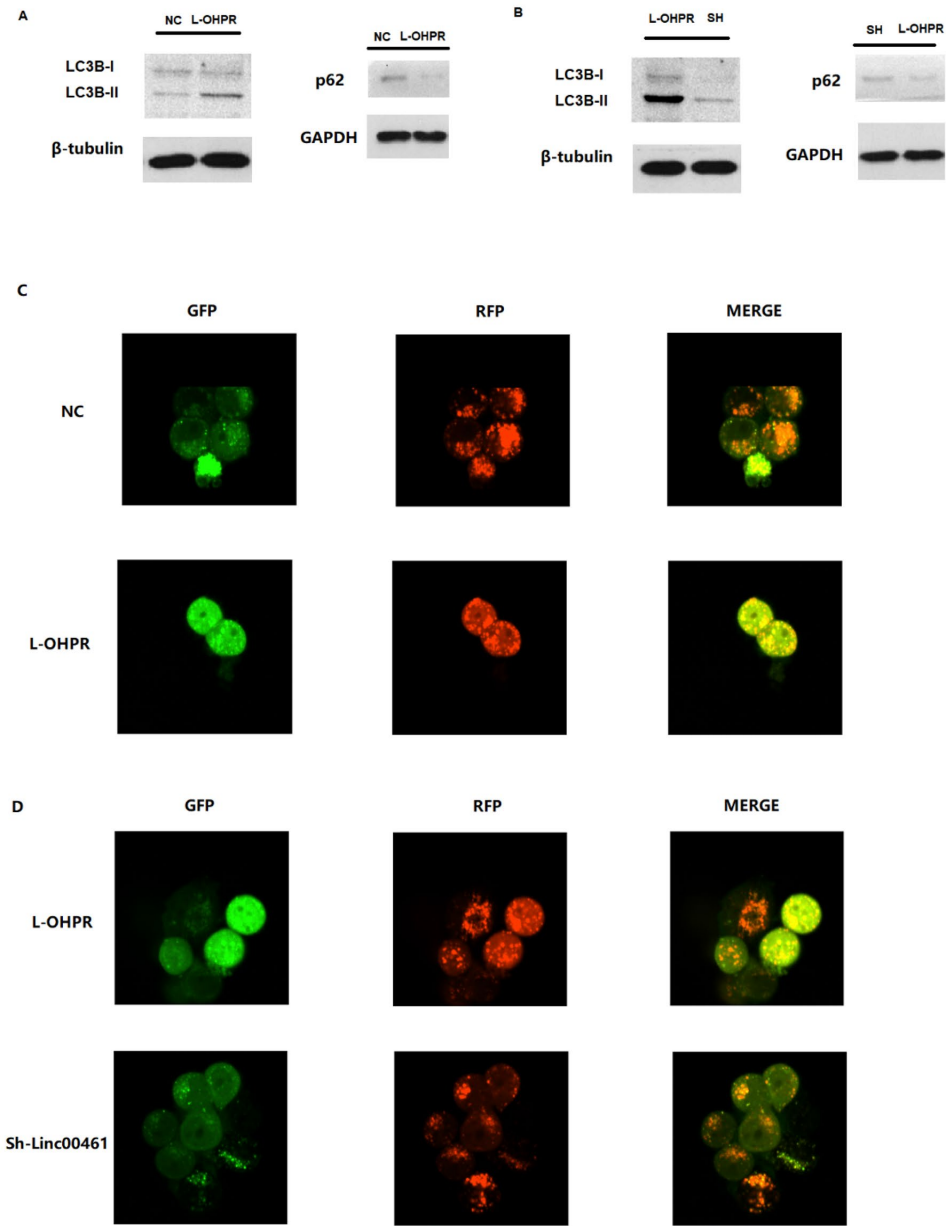
(**A**) Expression of LC3B and p62 in LOHPR SGC7901 cell line compared with SGC7901 cell line; the western blot for LC3B and p62 were cropped from different gels; (**B**) expression of LC3B and p62 in LINC00641 knock down LOHPR SGC7901 cell line; The western blot for LC3B and p62 were cropped from different gels; (**C**) we have transfected autophagy lentivirus into target cells and noticed that average green dot number was higher in L-OHPR cell line than that in SGC-7901(NC) cell line, indicated that autophagy was enhanced in L-OHPR cell line; (**D**) the average green dot number was lower in LINC00641 knock down SGC-7901 cell line, indicating that autophagy was inhibited.

LC3 II was highly expressed and the expression of LC3 I and p62 was lower in the L-OHP-resistant SGC7901 cell line (Fig. 5A). Therefore, we assumed that the autophagy process was involved in regulating L-OHP resistance. We then down-regulated the expression of linc00641 in the SGC7901 cell line and found that LC3 II expression was inhibited and LC3 I and p62 expression was enhanced (Fig. 5B). Thus, LINC00641 may induce the autophagy process to regulate L-OHP resistance. Furtherly, we transfected autophagy lentivirus into target cells and noticed that average green dot number was higher in L-OHPR cell line than that in SGC-7901(NC) cell line, indicated that autophagy was enhanced in L-OHPR cell line (Fig. 5C). Besides, we found that LINC00641 knock down would lead to inhibited autophagy in L-OHPR (Fig. 5D).

### Rescue experiments for LINC00641 and miR-582-5p interaction

**Figure 6:**
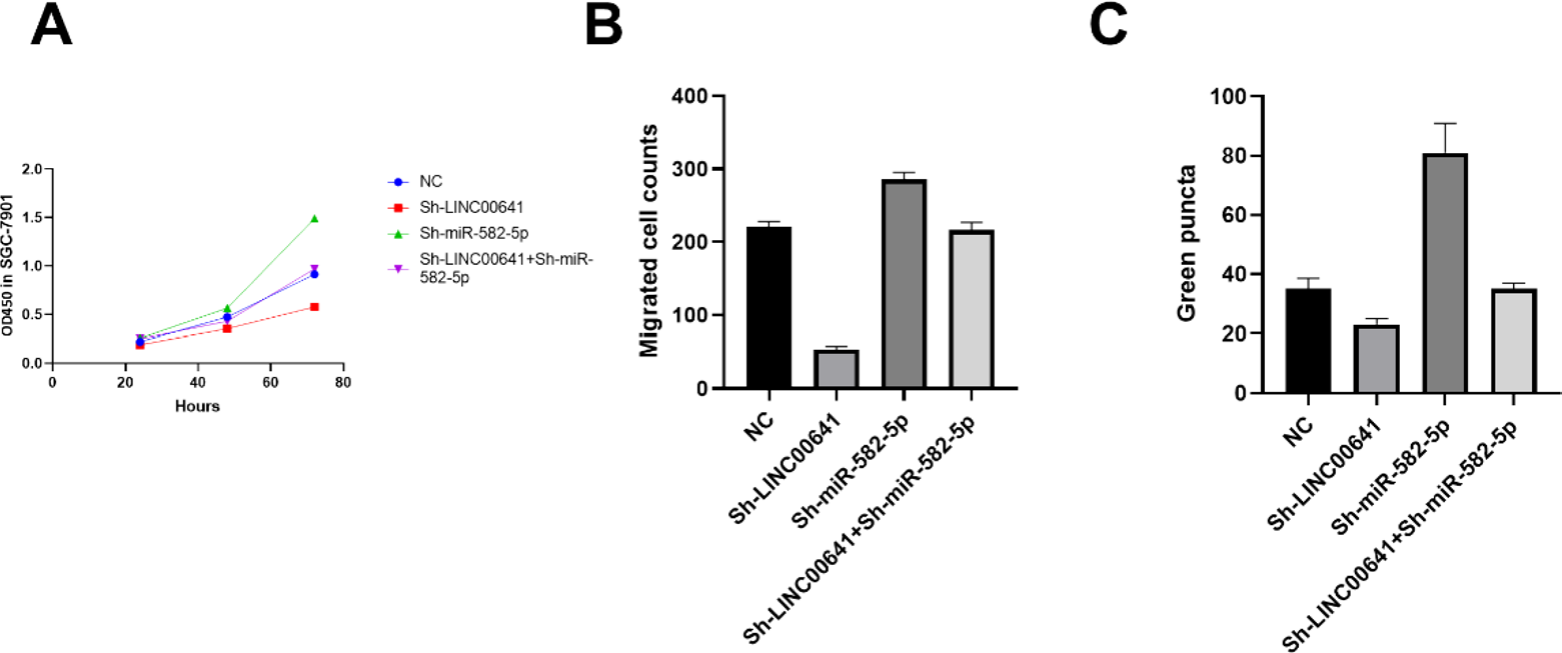
We have transfected negative control lentivirus, Sh-LINC00641, Sh-miR-582-5p and Sh-LINC00641 + Sh-miR-582-5p respectively into SGC7901 L-OHPR cell line. (**A**) miR-582-5p knock down can rescue the inhibitory effect of LINC00641 down-regulation on proliferation; (**B**) miR-582-5p knock down can rescue the inhibitory effect of LINC00641 down-regulation on migration; (**C**) miR-582-5p knock down can rescue the inhibitory effect of LINC00641 down-regulation on autophagy.

We have transfected negative control lentivirus, Sh-LINC00641, Sh-miR-582-5p and Sh-LINC00641 + Sh-miR-582-5p respectively into SGC7901 L-OHPR cell line. miR-582-5p knock down can rescue the inhibitory effect of LINC00641 down-regulation on proliferation (Fig. 6A) and migration (Fig. 6B). As shown in Fig. 6C, the green puncta in Sh-LINC00641 + Sh-miR-582-5p group is not significantly different from that in NC group, therefore, we can infer that LINC00641 can enhance the autophagy by suppressing the expression of miR-582-5p.

## Discussion

An increasing number of studies have indicated that lncRNAs are involved in cancer progression. In the present study, we found that LINC00641 was highly expressed in gastric adenocarcinoma and in L-OHP resistant gastric cancer cell lines. High LINC00641 expression was associated with a poor prognosis, which indicated that LINC00641 can be regarded as a biological indicator of poor prognosis. Inhibition of LINC00641 was observed to suppress cell viability and inhibit the autophagy process, which made gastric cancer cells more sensitive to L-OHP. Furthermore, miR-582-5p was proved to be a downstream target of LINC00641 in gastric adenocarcinoma cells using Starbase V3.0 (Supplementary Fig. 1). The qRT-PCR and western blot results demonstrated that LINC00641 exerted its biological function and activated autophagy by interacting with miR-582-5p. Based on these results, we suggest that LINC00641 and miR-582-5p are biomarkers for predicting overall survival; and they are involved in regulating L-OHP resistance by altering autophagy in gastric adenocarcinoma.

The induction of autophagy in gastric carcinoma cells during L-OHP treatment has been viewed as a protective mechanism, as it can help cells to make use of damaged organelles and recycle energy under stress caused by L-OHP. In this study, down-regulation of LINC00641 resulted in gastric carcinoma cells being more sensitive to L-OHP by inhibiting autophagy. Numerous studies have reported that lncRNAs can induce autophagy through various mechanisms. LncRNAs can function as ceRNA by binding microRNA to regulate autophagy-related genes. For example, lncRNA APF was reported to relieve transcriptional suppression of ATG7 by down-regulating miR-188-3p^13^. A previous study showed that LINC00641 promoted autophagy initiation by acting as a competitive endogenous RNA of miR-153-3p in a nutrient deprived environment^14^. LncRNAs can also influence autophagy-related genes or related signal pathways by binding important effector proteins. Ectopic expression of lncRNA HULC in hepatocellular carcinoma cells promoted autophagy initiation by protecting the Sirt 1 (silent information regulator 1) protein from ubiquitination by up-regulating ubiquitin-specific peptidase 22 (USP22)^15^. It was recently reported that lncRNA *NBR2* could bind and promote the activation of 5′ AMP-activated protein kinase (AMPK) which is a well-characterized molecule in autophagy regulation^16,17^. To gain insight into the clinical value of LINC00641 in gastric adenocarcinoma, we examined the expression of LINC00641 in 173 gastric adenocarcinoma tissues. The results indicated that LINC00641 was up-regulated in gastric adenocarcinoma and associated with a poor prognosis. It also played an oncogenic role in gastric adenocarcinoma. One study reported that LINC00641 was associated with extracellular matrix receptor interaction, focal adhesion, and mitogen-activated protein kinase signaling pathways in glioblastoma according to the WGCNA (weighted gene co-expression network analysis) algorithm. This result indicated the potential oncogenic functions of LINC00641 in tumor migration and invasion. Furthermore, autophagy initiation was involved in tumor motility. A previous study demonstrated that inhibition of autophagy could suppress cell migration and invasion. Autophagy can mediate the assembly and disassembly of the cell matrix, which significantly influenced cell migration^18^. In addition, we found that up-regulation of LINC00641 in gastric adenocarcinoma promoted cell migration in vitro and induced autophagy initiation under L-OHP treatment. However, it was shown that LINC00641 is a tumor suppressor in bladder cancer, as up-regulation of LINC0064 inhibited the progression of bladder cancer via the miR-197-3p/KLF10/PTEN/PI3K/AKT signal pathways^12^. Therefore, more studies are required to investigate the biological functions and molecular mechanisms of LINC00641 in a variety of cancers.

In conclusion, our study showed that LINC00641 and miR-582-5p are novel biomarkers in gastric adenocarcinoma patients for predicting overall survival. In addition, LINC00641 and miR-582-5p were associated with L-OHP resistance by regulating the autophagy process.

## Supplementary information

Supplementary Information.
