# Supplementary material for: LINC00641/miR-582-5p mediate oxaliplatin resistance by activating autophagy in gastric adenocarcinoma
Source: Sci Rep. 2020 Sep 11;10:14981. doi: 10.1038/s41598-020-70913-2 (PMC7486928; doi:10.1038/s41598-020-70913-2)

LINC00641/miR-582-5p mediate oxaliplatin resistance by activating autophagy in gastric adenocarcinoma

Yunfeng Hu [1,2], Yani Su [2], Xia Lei [3], Hong Zhao [2], Lelin Wang [4], Tian Xu [2], Jing Guo [2], Weiwei Yang [2], Xiaozhi Zhang [1]

1.Department of Radiation Oncology, The First Affiliated Hospital of Xi'an Jiaotong University, Xi'an, Shaanxi, China.

2.Department of Radiation Oncology, The First Affiliated Hospital of Yan’an University, Yan’an, Shaanxi, China.

3.Department of Gynecology, The First Affiliated Hospital of Yan’an University, Yan’an, Shaanxi, China.

4.Department of Thoracic surgery, The First Affiliated Hospital of Xi'an Jiaotong University, Xi'an, Shaanxi, China.

```
LINC00641     Target: 5' aggAAAUGGUGUCUUUGAACUGUAg 3'
                            || |||| |   : |||||||
miR-582-5p    miRNA : 3' ucaUUGACCA-ACUUG-UUGACAUu 5'
```

Supplementary Figure1 The binding site between LINC00641 and miR-582-5p based on Starbase

Variables	HR	95% CI of HR		P value
		Lower 95% CI	Upper 95% CI	
linc00461	1.462±0.221	0.949	2.254	0.085
miR-582	0.565±0.238	0.355	0.900	0.016
CEA	1.583±0.204	1.061	2.361	0.024
Age	1.689±0.211	1.116	2.557	0.013

Supplementary table1 COX model for linc00641, miR-582, CEA and Age in gastric cancer patients.

Western blot for LC3

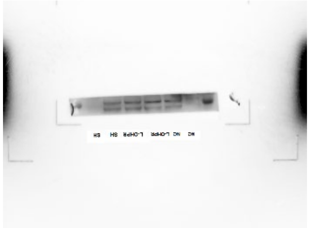

Western blot for β-tubulin

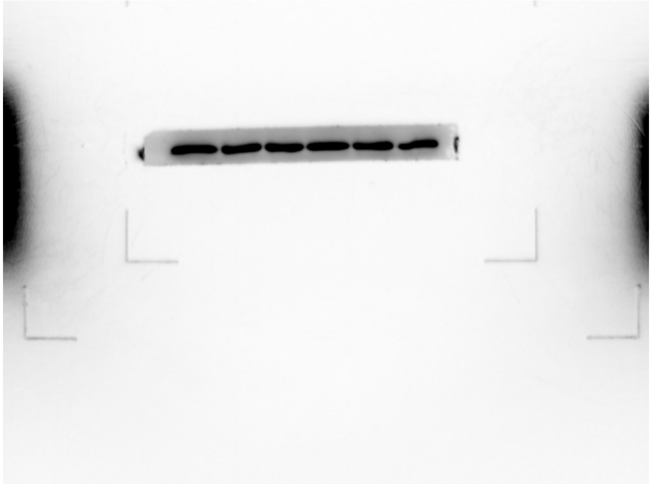

Western blot for GAPDH

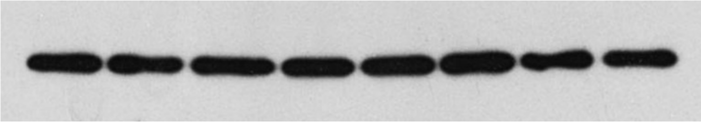

Western blot for p62

NC NC NC L-OHPR NC L-OHPR SH L-OHPR

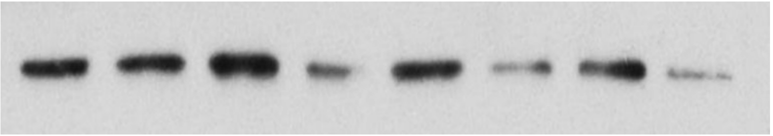

Supplement: Supplementary file 1 — Supplementary Information. [file 41598_2020_70913_MOESM1_ESM.pdf]
